# Observing Kelvin–Helmholtz instability in solar blowout jet

**DOI:** 10.1038/s41598-018-26581-4

**Published:** 2018-05-25

**Authors:** Xiaohong Li, Jun Zhang, Shuhong Yang, Yijun Hou, Robert Erdélyi

**Affiliations:** 1 CAS Key Laboratory of Solar Activity, National Astronomical Observatories, Chinese Academy of Sciences, Beijing, 100101 China; 2 School of Astronomy and Space Science, University of Chinese Academy of Sciences, Beijing, 100049 China; 3 Solar Physics and Space Plasma Research Centre, School of Mathematics and Statistics, University of Sheffield, Hicks Building, Hounsfield Road, Sheffield, S3 7RH UK; 4 Department of Astronomy, Eötvös Lorand University, Pázmány Péter sétány 1/A, Budapest, H-1117 Hungary

## Abstract

Kelvin–Helmholtz instability (KHI) is a basic physical process in fluids and magnetized plasmas, with applications successfully modelling e.g. exponentially growing instabilities observed at magnetospheric and heliospheric boundaries, in the solar or Earth’s atmosphere and within astrophysical jets. Here, we report the discovery of the KHI in solar blowout jets and analyse the detailed evolution by employing high-resolution data from the Interface Region Imaging Spectrograph (IRIS) satellite launched in 2013. The particular jet we focus on is rooted in the surrounding penumbra of the main negative polarity sunspot of Active Region 12365, where the main body of the jet is a super-penumbral structure. At its maximum, the jet has a length of 90 Mm, a width of 19.7 Mm, and its density is about 40 times higher than its surroundings. During the evolution of the jet, a cavity appears near the base of the jet, and bi-directional flows originated from the top and bottom of the cavity start to develop, indicating that magnetic reconnection takes place around the cavity. Two upward flows pass along the left boundary of the jet successively. Next, KHI develops due to a strong velocity shear (∼204 km s^−1^) between these two flows, and subsequently the smooth left boundary exhibits a sawtooth pattern, evidencing the onset of the instability.

## Introduction

KHI, as proposed by Lord Kelvin in 1871^1^ and Helmholtz in 1868^2^, occurs when two parallel fluids with different velocities flow alongside each other, with a shear exceeding a critical value^3^. KHI has been shown to be important to understand a considerable number of astrophysical and space physical phenomena such as the dynamic structure at cometary tails^4^, relativistic outflows and oscillations in astrophysical jets^5,6^, the merger of neutron star systems^7^ or the transfer of energy and momentum between the solar wind and solar system’s planetary magnetospheres^8–11^. KHI may also play a very important role in plasma heating as it develops small scales therefore enhancing the dissipative processes, such as turbulent viscosity^12^. High-resolution observations with modern space instruments enable us to detect the KHI in the Sun, e.g. at the boundaries of lager-scale coronal mass ejections (CMEs)^13–16^ or within fine-scale structures (e.g. filaments^17^) in the solar corona^18^.

Solar jets^19^ occur over a wide range of scales in the solar atmosphere from spicules^20^, anemone jets^21^, to network jets^22^, X-ray jets^23,24^, etc. Often they are localised plasma eruptions likely to develop at e.g. magnetic reconnection areas where new emerging flux reconnects with pre-existing fields which have opposite magnetic polarities^25^. A blowout model^26^ has been proposed to explain the formation of jets, i.e. the strong shear and twist of the magnetic field in the core of the jet’s arch drive an ejective eruption, producing blowout jets^27,28^. We have observed KHI in several solar jets, and here we establish the most distinct and most detailed example observed by IRIS^29^.

The IRIS spacecraft yields simultaneous spectral and imaging observations of the solar atmosphere. On 12 June 2015, there were 8 active regions (ARs) on the solar disk. One of them, AR 12365, was located southeast of a trans-equatorial coronal hole. At the boundary of the main sunspot of this AR, several jets occurred successively. IRIS observed AR 12365 with a pixel size of 0″33 and a cadence of 3 seconds. The high temporal resolution enables us to study the evolution of the jets in unprecedented details. We also employ the associated magnetograms taken by the Helioseismic and Magnetic Imager (HMI)^30^ and the concurrent multi-wavelength images sampled by the Atmospheric Imaging Assembly (AIA)^31^ on-board the Solar Dynamics Observatory (SDO)^32^ to examine the magnetic and velocity field evolution of the AR, in particular, the dynamics of the jets present therein.

**Figure 1:**
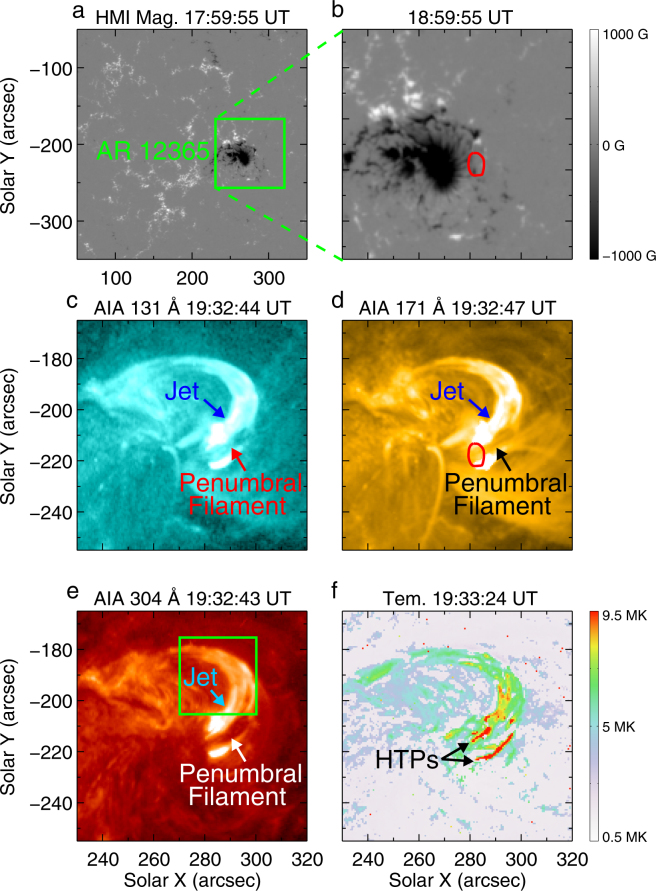
Overview of the jet observed by SDO. (**a,b**) HMI line-of-sight (LOS) magnetograms displaying the magnetic field environment of the jet footpoint. The green box in (**a)** outlines the field-of-view (FOV) of (**b** to **f)**. The red contour in (**b)** represents the footpoint of the jet, which is determined from AIA observations in (**d**). (**c–e**) AIA 131 Å, 171 Å and 304 Å images displaying the appearance of the jet at its peak evolution time. The green box in (**e**) outlines the FOV of Fig. 3. (**f**) plots the temperature derived from the wavelengths of 94 Å, 131 Å, 171 Å, 193 Å, 211 Å and 335 Å. The black arrows indicate the hottest temperature patches (HTPs) in the jet.

**Figure 2:**
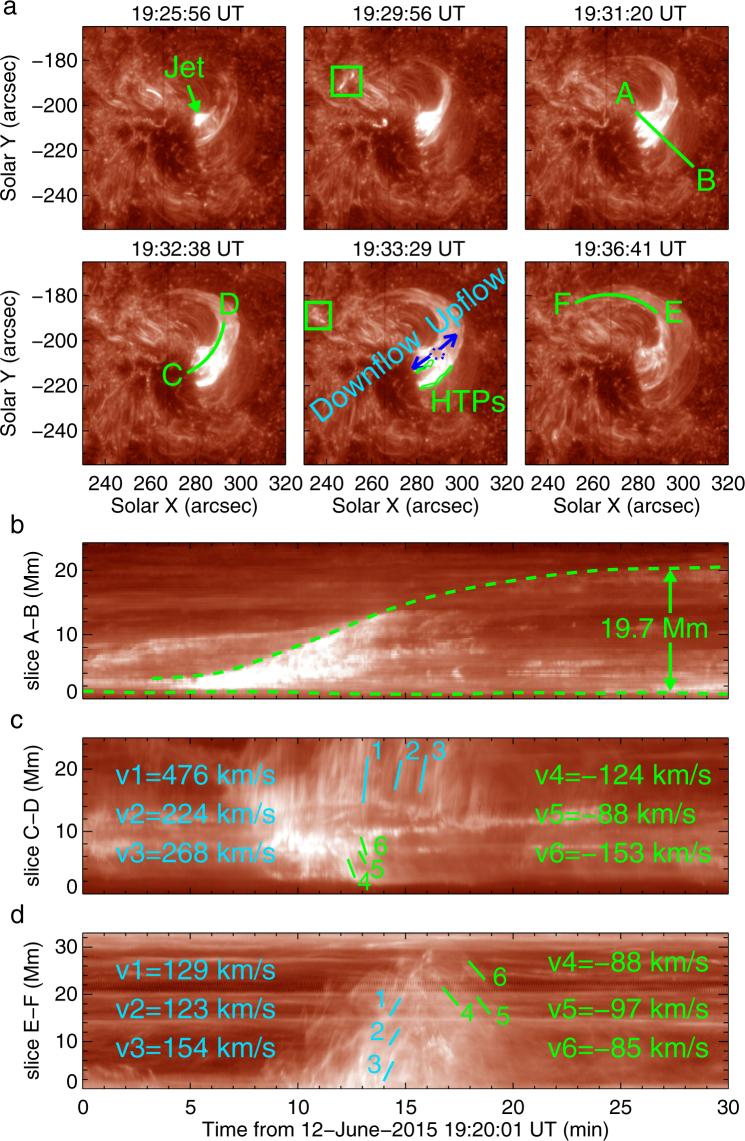
Evolution of the jet observed by IRIS 1400 Å. (**a**) IRIS 1400 Å slit-jaw-images (SJIs) displaying the development of the jet. The green boxes in the second and fifth panels outline the brightenings. The blue dotted curve in the fifth panel denotes a “cavity” within the jet. The green line AB shows the cross-cut position used to obtain the stack plot which displays the jet’s width variation over time, as shown in (**b**). The green dashed lines denote the outer boundary of the jet that reached 19.7 Mm in height by the end. (**c**) Evolution at the position of curve CD (see panel at 19:32:38 UT in **a**) which passes through the cavity and the bi-directional flows (see panel at 19:33:29 UT in **a**). The velocities of selected representative bright structures are displayed. (**d**) Temporal evolution of flows at the position of curve EF (see panel at 19:36:41 UT in **a**). The flows from right to left (1−3 in cyan color) and from left to right (4−6 in green color) are shown in this plot.

Figure 1 shows the photospheric magnetic field of the AR and the jet in EUV observed by SDO on 12 June 2015. The positive magnetic flux (visualised by white color) emerged at the western boundary of the main negative polarity (dark colored) sunspot of the AR (Supplementary Video 1). At the magnetic reconnection region, below which the emerging positive flux cancelled out with the pre-existing negative fields, a jet appeared at 19:24 UT, and reached its emission peak at about 19:32 UT. A filament, which was anchored in the penumbra of the main sunspot with negative polarity, was involved in the jet and filament material was ejected upwards. This process was clearly detected in both higher and lower temperature wavelengths, e.g., 131 Å, 171 Å and 304 Å (Fig. 1c–e and Supplementary Video 2). The footpoint of the jet is shown in Fig. 1d (red circle) and is also overlaid on the corresponding magnetogram (Fig. 1b). There were concurrent brightenings in the jet tail as shown in Fig. 2a (see the green boxes at 19:29:56 UT and 19:33:29 UT). During its evolution, the jet expanded with the fastest broadening taking place from 19:30 UT to 19:35 UT, reaching its widest width of 19.7 Mm (Fig. 2b). A cavity structure above the jet’s base appeared during this broading period (denoted by blue dotted circle in Fig. 2a and Supplementary Video 3). Meanwhile, there were rapid bi-directional flows detected from the cavity. The velocities of the upflows were about 224–476 km s^−1^ and the downflows velocities were 88−153 km s^−1^ (Fig. 2c). Such bi-directional motion was also found in a rather different context by e.g.^33^. Comparing the temperature map (Fig. 1f) derived from AIA observations with IRIS images (Fig. 2a), we find that the temperature near the cavity was much higher, nearly 15.4 MK. All these rather detailed features suggest that magnetic reconnection took place around the cavity, and that magnetic energy was released resulting in changes of the magnetic topological structure near the jet’s base. After the peak stage of the jet formation, there were backflows from the tail of the jet, with velocities of ∼90 km s^−1^ (see Fig. 2d, denoted by v4−v6) in the direction perpendicular to the line of sight. The Doppler blueshift velocity of the backflows measured by the spectra data is about 10.3 km s^−1^ (see Supplementary Fig. 1). The jet also displayed clockwise rotation seen from the jet’s base (see Methods and Supplementary Fig. 2 for details).

**Figure 3:**
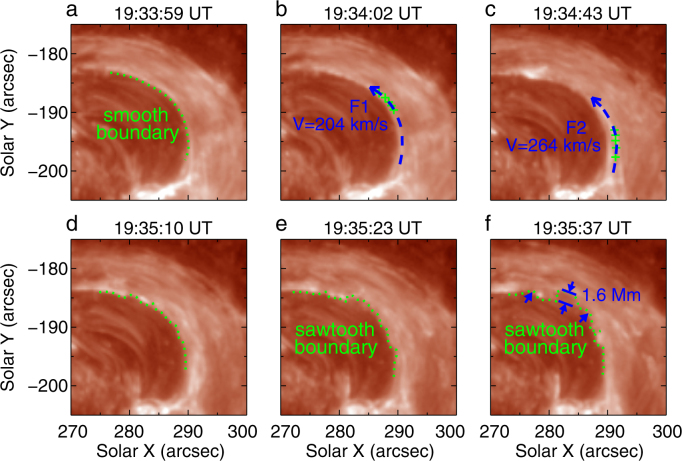
IRIS 1400 Å SJIs showing the KHI detected at the left boundary of the jet. The green curves in panels a and d–f denote the left boundary which changes from being smooth (**a**) into a sawtooth pattern (**d–f**). The blue curves in (**b** and **c)** denote the trajectories of the first (F1) and the second (F2) flows, respectively. The blue arrows indicate the directions of “F1” and “F2.” The green crosses in **b** (**c**) show the trajectory of a bright point in “F1” (“F2”) which we track to determine the velocity of “F1” (“F2”), with the value of 204 km s^−1^ (264 km s^−1^). The blue arrows in (**f)** display the distortions of the boundary, with the largest distortion being 1.6 Mm.

**Figure 4:**
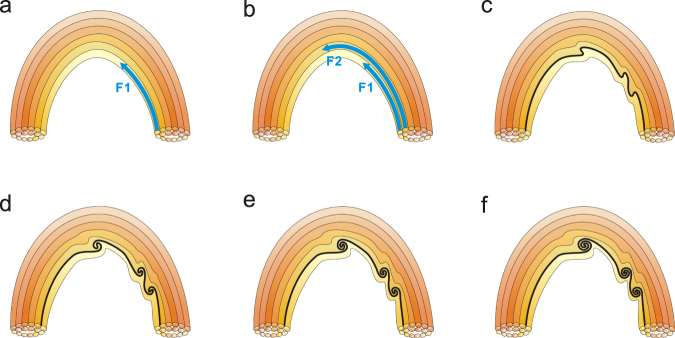
The development of the KHI in the flux tubes of the jet. Individual flux tubes are distinguished with different colors. The skyblue arrows in panels a and b represent the flows in the flux tubes. Panels c–f show the change of the boundary and the development of vortices at the interface after the KHI developed.

Let us now focus on the formation of the KHI. Fig. 3 and Supplementary Video 4 show the development of the KHI in the jet. The left boundary of the jet was very smooth (Fig. 3a) initially. At about 19:33:15 UT (see Supplementary Video 4), a thin layer of bulk motion “F1” with an estimated thickness of 630 km, originating from the jet’s base, passed along the left side of the jet for about an extent of 20 Mm. We employ the method of tracking the movement of a small bright point in “F1” and determine the velocity of “F1” (see Methods for details) to be about 204 km s^−1^ (Fig. 3b). Some 80 seconds later, another thin flow strip, labelled as “F2” with a width of 460 km, also passed along the left side of the jet, on the right of and close to “F1.” The “F2” velocity was estimated 264 km s^−1^ (Fig. 3c), meanwhile “F1” slowed down to 60 km s^−1^. Thus, a strong velocity gradient (i.e. shear) of about 204 km s^−1^ has formed between these two flow streams. Assuming that the jet consists of an ensemble of thin magnetic flux tubes^34^, we draw a cartoon to describe the development of the KHI (see Fig. 4). As shown in Fig. 4a and b, the plasmas in the flux tubes, with different densities, flow alongside each other with different velocities, causing the onset of the KHI. Vortices form at the interface between these two flux tubes, and the boundary of the flux tubes become distorted (see Fig. 4c–f). Based on the theoretical consideration (see Methods for details), we estimate that the KHI could happen with a velocity difference of 204 km s^−1^. In deed, the KHI did manifest as the left boundary of the jet became distorted, which is demonstrated in Fig. 3d−f. The boundary turned into a sawtooth-shape as shown in Fig. 3f, with a maximum distortion of 1.6 Mm. This vortex-shaped boundary surface is an important feature when the KHI takes place^35^. The growth rate of the KHI is measured to be approximately 0.063, the same order of magnitude as the theoretical value (see the explanations in Methods and Supplementary Fig. 3 for details). Considering the distance between two vortices as the characteristic length scale for the associated wavelength (*λ* ∼ 5000 km), and half of the flow widths as the estimated boundary layer thickness (a ∼ 545 km), then the wavelength satisfies the condition of the fastest growing KH mode given by *λ* = (2 − 4) × *π* × a^36^, that is another verification for KHI really taking place. During the evolution of the KHI, the temperature of the plasma where the KHI occurred increased by approximately 2 MK (see Supplementary Fig. 4 and Methods). When the KHI is just manifested, we find that the material at the left boundary of the jet became rotating in the direction contrary to that of the jet (Supplementary Fig. 5). By tracking some small bright points, we have determined the rotation diameter ∼1800 km, with an angular velocity of 18° s^−1^ and projected velocity of 180 km s^−1^.

Here, we clearly see and report that two upward flows, with a strong velocity shear of 204 km s^−1^, travelling parallel to each other, drive the onset of the KHI in a blow-out jet in the solar atmosphere. The high spatio-temporal resolution capability from IRIS enable us to actually detect the developing process (80 seconds, see Fig. 3b,e) and the distortion scale (⩽1.6 Mm, ∼2 arcsec) of the KHI. The formed jet occurs in a region possessing stronger magnetic fields, while the solar wind and solar filaments in which the KHI has previously been detected have considerably weaker magnetic fields. Our finding extends evidently the range where the KHI takes place in the solar atmosphere to much smaller scales, and implies that the KHI may be rather ubiquitous on the Sun in the presence of jets. This latter point is a very important one, as the smaller the scales where the KHI may take place, the more important this instability may be for the heating of the localised plasma^37^. So far, the lack of evidence of small-scale KHI vortices may be related to the insufficient resolution. Considering that the KHI is undisputedly an important aspect in (magneto)hydrodynamics, so the study of KHI is conducive not only for understanding solar activities, but also to comprehending fundamental physical processes in fluids and magnetic plasmas. Theoretically, there may be also other possibilities, for example, the sawtooth boundary is the manifestation of blob formation or LOS effects of twist structures, but these explanations would require more details to support them.

## Methods

### Instrumentation and data

1400 Å SJIs are obtained from the IRIS spacecraft with a cadence of 3 seconds, and a pixel scale of 0. ″33. For the Doppler velocity measurement, we use data from spectrograph on IRIS in 1402.77 Å. In addition to this, EUV images from the AIA on board the SDO are employed to display the dynamics of the jet. The cadence is 12 s and the spatial sampling is 0. ″6 pixel^−1^. We use data observed in 304 Å, 171 Å, 193 Å, 211 Å, 131 Å, 335 Å and 94 Å, which have strong responses to logarithmic temperatures of about 4.7, 5.8, 6.2, 6.3, 7.0, 6.4 and 6.8 Kelvin, respectively. The LOS magnetograms with 45-sec cadence and 0. ″5 per pixel from HMI on SDO are also applied in order to study the magnetic evolution.

### Standard jet model

The standard jet model was put forward some decades ago^38^. The key idea of this model is that a current sheet is formed as the jet-base magnetic arch approaches the ambient opposite-polarity open field. Magnetic reconnection takes place when the current sheet becomes sufficiently strong and thin. The current sheet is demolished due to a burst of interchange reconnection between the jet-base magnetic arch and its ambient field. Magnetic reconnection releases energy and the plasma in the reconnected field is heated to several million degrees, hot enough to emit X-rays. Some hot plasma may escape along the reconnected open field lines and may become the spire of the jet. Meanwhile, a new magnetic arch is formed and may shrink downward with the reconnection-heated hot plasma in it, which is observed as a bright point in X-ray images^39,40^.

### Blowout jet model

According to the blowout jet model^26^, the jet-base magnetic arch approaches the ambient opposite-polarity open field with intense shear and twist at the core field in the base arch. The strong shear and twist of the magnetic field in the core of the jet’s base arch drive an ejective eruption, generating blow-out jets and associated waves^28^. The onset of blowout jets is caused by a burst of reconnection at the interface current sheet, in the same manner as for standard jets. Then, the sheared-core arch begins to erupt and drives more breakout reconnections. Reconnection may occur at three different locations: at the interface current sheet, between the opposite-polarity stretched legs of the internal erupting field, and, between the leg of the erupting core-field flux rope and its opposite-polarity ambient open field. The blow-out eruptions continue to build the jet’s bright point, may generate a flare arcade inside the base arch, and may result in a spire with complex and highly magnetically stressed structure^41–44^. Based on the blowout model, a cartoon is drawn to display the reconnection scenario of the jet in the text (see Supplementary Fig. 6). The first drawing is the magnetic topological structure before the jet. The ambient field is complex, and only a few representative field lines are drawn with yellow color in order to visualise the connections. The red pattern represents the cold filament material. New magnetic flux, depicted with blue lines, are emerging constantly and approaching the ambient field. Reconnections occur at different places and the magnetic structure after the onset of the jet is shown in Supplementary Fig. 6b. The filament is activated and is ejected out as displayed by the red contour. After the onset, the reconnections continue to take place and drive the spectacular evolution of the jet.

### Untwisting in the solar jets

The helical structure, or spinning, is a common geometrical feature of solar jets and many studies reveal that it is driven by magnetic untwisting^45^. In a blowout jet, there is a twisted flux rope (or filament) in the jet’s base and the twist may be released by the reconnection between the flux rope and ambient open fields, thus leading to the magnetic untwisting^46^. Here, the rotation of the jet may be caused by the magnetic untwisting as well. The pitch angle is about 30°−60°. Assuming that the plasma moves along the magnetic line and the trajectory represents the morphology of the magnetic field, we estimate that the ratio of the azimuthal component with regard to the component along the magnetic axis is approximately 0.6−1.7.

### KHI in the solar atmosphere

KHI was first observed at quiescent prominences in the solar atmosphere by using data obtained by the Solar Optical Telescope (SOT) on-board Hinode^17,18^. With the observations by SDO/AIA, KHI has been detected at the interface between a dimming area and the surrounding corona^47^ and on the flank of a fast CME less than 150 Mm above the solar surface^15^. KHI is mostly identified by the appearance of growing ripples or the associated plume head vortex. The kink-like oscillation of a streamer^48^ and rapid redshifted and blueshifted excursions^49^ in the solar chromosphere are presumed to be caused by the KHI as well.

### Magnetic flux estimation

We estimate the magnetic flux at the footpoint of the jet using photospheric magnetic field obtained by HMI. According to the conservation of magnetic flux within the same group of magnetic loops, one can work out the magnetic flux density of other positions of the jet in case of knowing its area. We assume that the body of the jet is a cylinder, so its cross-sectional area is easy to obtain since we can measure the diameter.

### Velocity measurement

In order to derive the velocities of the bulk flows, we choose some representative distinct bright points in the flows and trace their movement. The cadence allows us to measure the distance moved every 3 seconds and the total time and total distance can be determined easily. Dividing distance by time, one obtains the average velocity.

### Temperature measurement

Using the differential emission measure (DEM) analysis method which is based on the “xrt_dem_iterative2.pro” in the Solar Software package^50,51^, we derive the temperature map from the AIA 94 Å (Fe XVIII), 131 Å (Fe VIII, Fe XX, Fe XXIII), 171 Å (Fe IX), 193 Å (Fe XII, Fe XXIV), 211 Å (Fe XIV) and 335 Å (Fe XVI) observations. As there is little temperature discrimination at the low temperatures in the AIA channels, we have to mention that this method for temperature measurement is only applicable for coronal structures hotter than ∼1 MK. In this letter, the jet is visible simultaneously in multiple AIA channels, which implies that the structure is either broadly multi-thermal, or at a cool (∼10^5^) temperature^52,53^. Since all AIA channels and the IRIS channel have a cool-temperature response due to cool, relatively weak spectral lines present in the passbands, and the jet is also clearly observed in the IRIS 1400 Å passband, it is more likely that the jet is Transition Region structure. Also, there exists much cooler filament material in the jet, so the DEM method may bring large error when calculating the temperature. Up to now, there is no better temperature measurement method that one can use, and the temperature value provided here is just for reference.

### Spectral analysis

We employ the IRIS raster level 2 data, which have been dark corrected, flat fielded, and geometrically corrected, to measure the associated Doppler shifts. We mainly analyze the emission of the Si IV 1402.77 Å line, which is formed in the middle transition region with a temperature of ∼0.065 MK. The nearby S I 1401.51 Å line is used for the absolute wavelength calibration^54^. We measure the Doppler shifts of the S I line for seven times (around the moment we focus) and compare the values with the standard one. The average different value is regarded as calibration value and the standard deviation is calibration error. To obtain the Doppler shift of the jet material, we apply a single-Gaussian fitting to the Si IV line profile (see Supplementary Fig. 4). At 19:34:12 UT, a bulk flow from the jet’s base to the tail emerged across the slit, as shown in Supplementary Fig. 4a. By analyzing the spectral profiles of Si IV 1402.77 Å, we find the Doppler redshift relative to the background was 21.8 km s^−1^ (Supplementary Fig. 4b,c). The wavelength calibration error is 4.4 km s^−1^. Using the same method, let us now analyze the motion of the backflow as it passed through the slit (see Supplementary Fig. 4d−f). The backflow progressed along the opposite direction to the original bulk flow and showed a clear Doppler blueshift, corresponding to an associated velocity of − 10.3 km s^−1^. The wavelength calibration error is 4.8 km s^−1^. The calibration error would change the Doppler velocity value of the flow. Comparing the calibration error with the Doppler velocity, we conclude that whether the flow is redshifted or blueshifted won’t be changed.

### Theoretical consideration

As mentioned above, the jet is assumed to be a group of thin magnetic flux tubes, and “F1” and “F2” are the flows in two adjacent flux tubes, respectively. In the following, we also denote by “F1” and “F2” the two flux tubes with bulk flows and their properties are distinguished with subscript “1” and “2”. To model the rather complex situation, we assume the plasma is uniform, incompressible, ideal and we only consider 2-dimension motion of the flux tube. At the footpoint of “F1” (“F2”), the magnetic field intensity is about 434 G (329 G) and is derived from the HMI LOS magnetogram. The flux tubes have diameters of 100 km at the photosphere. At the location where the KHI developed, the widths of the flows which have been measured above (630 km for “F1” and 460 km for “F2”) can be interpreted as the diameters of the flux tubes. According to the conservation of magnetic flux, therefore, the magnetic field intensities of “F1” and “F2” are approximately *B*_1_ = 11 G and *B*_2_ = 15 G. Using the DEM method, we obtain the total emission measure (*EM*) and the particle number density *n* can be derived using *n* = $\sqrt{EM/l}$ where the *l* is the depth of the flux tube. Assuming that *l* = 500 km, the total *EM*_1_ of 8.1 × 10^28^ cm^−5^ corresponds to a number density (*n*_1_) of 4.0 × 10^10^ cm^−3^ and the total *EM*_2_ of 1.0 × 10^29^ cm^−5^ corresponds to a number density (*n*_2_) of 4.5 × 10^10^ cm^−3^. Let us suppose that the plasma density in each tube is homogeneous, the KHI occurs if

1 $${(\overrightarrow{k}\cdot {\overrightarrow{V}}_{1}-\overrightarrow{k}\cdot {\overrightarrow{V}}_{2})}^{2} > ({\rho }_{1}+{\rho }_{2})[(\overrightarrow{k}\cdot {\overrightarrow{B}}_{1}{)}^{2}+{(\overrightarrow{k}\cdot {\overrightarrow{B}}_{2})}^{2}]/{\mu }_{0}{\rho }_{1}{\rho }_{2}$$

where $\overrightarrow{k}$,$\overrightarrow{V}$, $\overrightarrow{B}$, *ρ* are the wave vector, velocity, magnetic intensity and mass density in the flux tube, respectively. Presuming that $\overrightarrow{k}\parallel {\overrightarrow{V}}_{1}\parallel {\overrightarrow{V}}_{2}\parallel {\overrightarrow{B}}_{1}\parallel {\overrightarrow{B}}_{2}$, then we can work out that the velocity difference threshold is

2 $${\rm{\Delta }}V=|{\overrightarrow{V}}_{1}-{\overrightarrow{V}}_{2}|=\sqrt{({\rho }_{1}+{\rho }_{2})({B}_{1}^{2}+{B}_{2}^{2})/{\mu }_{0}{\rho }_{1}{\rho }_{2}}\mathrm{.}$$

Since *ρ* = *n* ⋅ *m* (*m* = 1.673 × 10 ^−27^ kg), and *B* and *n* have been already estimated, we obtain that the threshold Δ*V* = 279 km/s. As seen from the formula, the effect of the magnetic field on the KHI depends on its orientation. The magnetic field component parallel to the interface discontinuity can exert a restoring force, and can suppress the growth of the KHI, while the perpendicular component makes no difference^55^. Here, the magnetic field is not strictly parallel to the flow, as we observe that the second flow rotates as it passes though the side of the jet. Theoretically speaking, in this case, the KHI will occur as long as the angle between the flow and magnetic field is more than 43°, which is possible in our situation. These hypotheses are ideal and the results highly depend on the angle between the flow and magnetic field. However, the actual situation is complex and the twisting flux tube may lower the threshold of the angle. Recent three-dimensional (3-D) magnetohydrodynamics (MHD) simulations have indicated that although the draping of strong tailward component of the Parker-Spiral Interplanetary Magnetic Field tends to stabilize the growth of the instabilities, KHI with a titled $\overrightarrow{k}$ oriented approximately 41° from the shear flow plane still develops into the nonlinear phase and the magnetic reconnection may occur as a secondary effect^56^. The simulations help to understand how the KHI happens indeed for solar jets.

### Growth rate of the KHI

Once the KHI takes place, we choose two positions (see Fig. 3f where the distortion is the largest and the place on its right indicated with blue arrow) on the jet and measure these distortions. The distortions over time are plotted in Supplementary Fig. 6. The distortions are exponential growths and the growth rates are about 0.059 and 0.067. In theory, the growth rate is

3 $$\begin{array}{rcl}\gamma  & = & \sqrt{{\rho }_{1}{\rho }_{2}{(\overrightarrow{k}\cdot {\overrightarrow{V}}_{1}-\overrightarrow{k}\cdot {\overrightarrow{V}}_{2})}^{2}-({\rho }_{1}+{\rho }_{2})[(\overrightarrow{k}\cdot {\overrightarrow{B}}_{1}{)}^{2}+{(\overrightarrow{k}\cdot {\overrightarrow{B}}_{2})}^{2}]/{\mu }_{0}}/({\rho }_{1}+{\rho }_{2})\\  & = & 2\pi \sqrt{{\rho }_{1}{\rho }_{2}{({\rm{\Delta }}V)}^{2}-({\rho }_{1}+{\rho }_{2})({B}_{1\parallel }^{2}+{B}_{2\parallel }^{2})/{\mu }_{0}}/\lambda ({\rho }_{1}+{\rho }_{2}\mathrm{).}\end{array}$$

Assuming that the angle between the flow and magnetic field is about 45°, with regard the distance between two vortices as the associated characteristic wavelength (∼5000 km), and substituting the value of the velocity difference into the formula, we can estimate the theoretical growth rate γ = 0.033. As the angle between the flow and magnetic field increases to 60°, the growth rate will rise to 0.093. Remarkably, the measured values are of the same order of the theoretically estimated values. The deviation may be caused by many reasons. In addition to the measurement error, the assumptions in the theory will also be accountable for the difference.

### Plasma heating of the KHI

We study the temperature variation and find that a heating process appears during the KHI as shown in Supplementary Fig. 4a. We choose an area (outlined by the black curve) and calculate the average temperature, which is about 2 MK hotter during the KHI than before and after the event. Theoretical studies have shown that the KHI on small scales can play an important role in the energy dissipation of the associated waves and jets, and in the plasma heating in the solar atmosphere. Here the temperature increase is thought to be caused by plasma heating process, which is induced by the KHI. The plasma heating associated with the KHI has been studied before in other astrophysical phenomena. Combining Cluster observation and MHD numerical simulations, a recent study provides evidence that ion-scale (200−2000 km) fast magnetosonic waves, generating inside the fluid-scale (36000 km) KH vortices at the Earths magnetospheric boundary, have sufficient energy to provide significant ion heating (2 keV = 20 MK increase for the observed ion flux population), thus demonstrating how the cross-scale energy transfer from fluid to ion scales can result in ion heating^57^. This cross-scale mechanisms may also contribute to the heating of the solar corona and play a role in other astrophysical plasmas^37^. Further, nonlinear 3-D MHD simulations have shown that the resonant dissipation layers are subject to the KHI, and these resonant layers generate small scale turbulent motions that enhance the dissipation parameters e.g. eddy viscosity, leading to the dissipation of energy and ultimately causing heating of the solar corona by Alfvén waves^58,59^. This scenario is a rather viable heating mechanism. The exact heating mechanism in ours reported here needs to be further examined at a much higher spatial and temporal resolution.

### Data availability

All the data used in the present study are publicly available. The IRIS data that support the findings of this study are available from http://iris.lmsal.com/. The SDO/HMI LOS magnetograms and the SDO/AIA EUV images can be downloaded from http://jsoc.stanford.edu/.

## Electronic supplementary material

Supplementary Video 1

Supplementary Video 2

Supplementary Video 3

Supplementary Video 4

Supplementary Video 5

Supplementary Information
