# Supplementary material for: Observing Kelvin–Helmholtz instability in solar blowout jet
Source: Sci Rep. 2018 May 25;8:8136. doi: 10.1038/s41598-018-26581-4 (PMC5970241; doi:10.1038/s41598-018-26581-4)
Supplement: Supplementary file 6 — Supplementary Information [file 41598_2018_26581_MOESM6_ESM.pdf]

Supplementary Information:

Observing Kelvin−Helmholtz instability in solar blowout jet

Xiaohong Li, Jun Zhang, Shuhong Yang, Yijun Hou & Robert Erdélyi

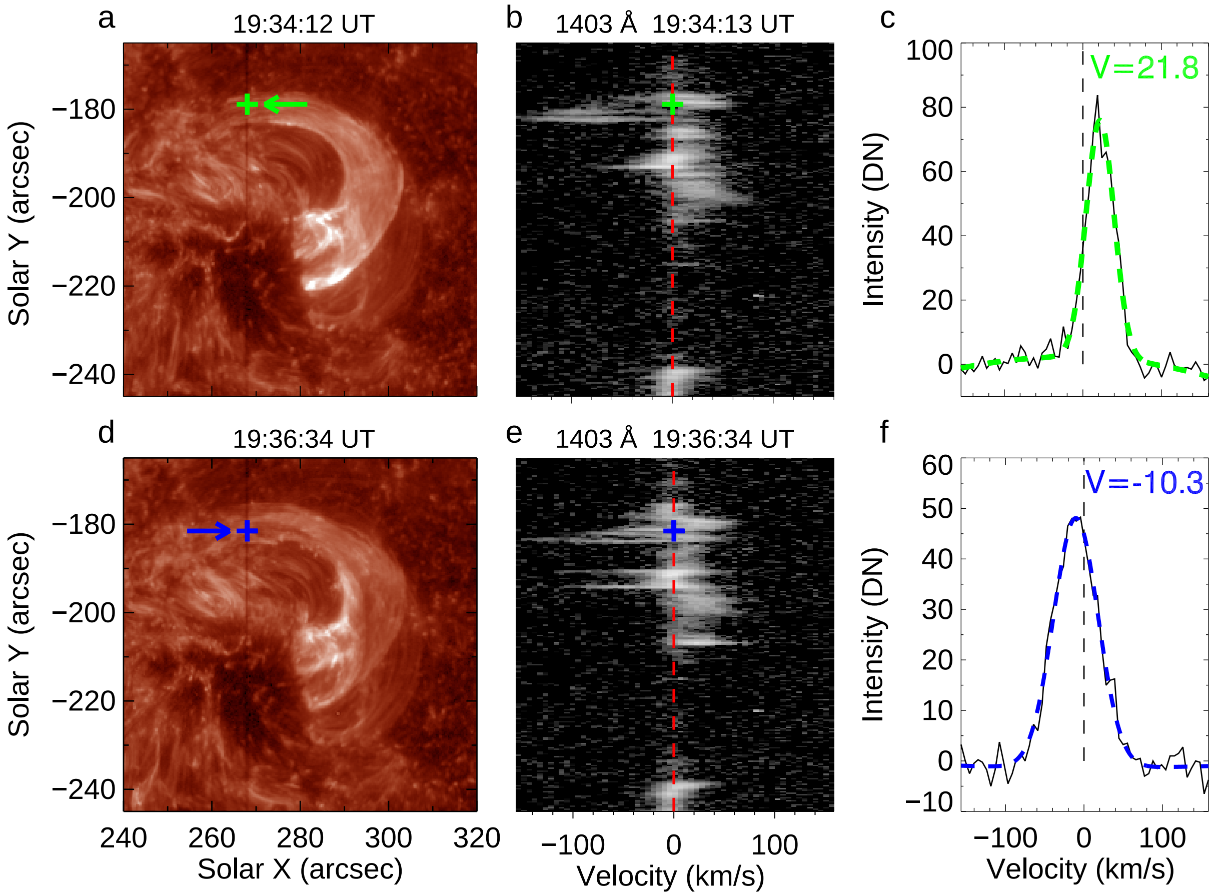

Supplementary Figure 1 | The IRIS 1400 Å images and Doppler velocity plots of the jet. **a** and **d** show IRIS 1400 Å SJIs displaying the jet at the given time. The green and blue symbols denote the positions where Doppler shifts are measured. The green and blue arrows indicate the directions of the flows at the target positions. **b** and **e** are simultaneous appearances of the Si IV 1403 Å spectra in the slit range of **a** and **d** for Doppler velocities from -160 km s^{-1} to 160 km s^{-1}. **c** and **f** exhibit observed profiles (black solid curves) and the corresponding single-Gaussian fittings (dashed curves) at the selected locations in **b** and **e**.

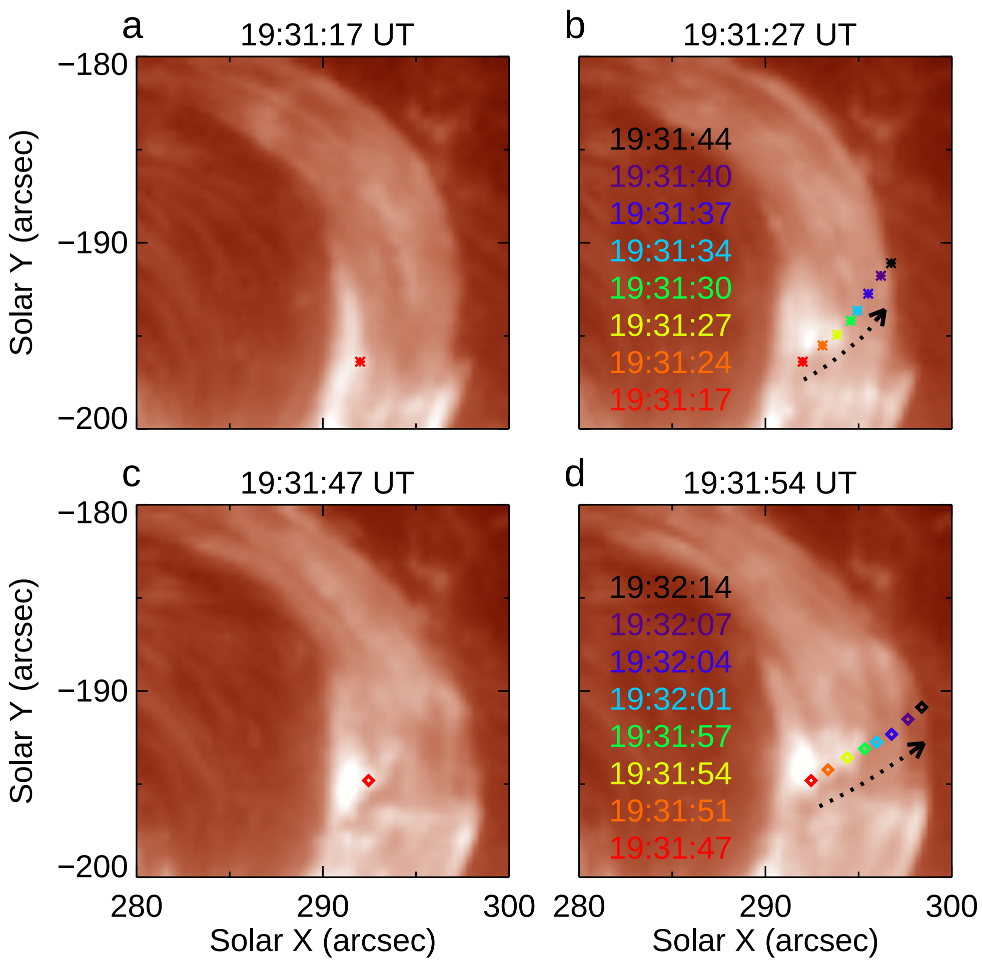

Supplementary Figure 2 | IRIS 1400 Å images displaying the rotation of the jet. **a** and **c** with red symbols show the bright points which we track. **b** and **d** highlight the positions of the tracking points indicated by the same symbols. The time of each position is denoted by the same symbol color and the arrows display the directions of motion. The average angular velocity is 6° s^{-1} and the projected velocity is about 210 km s^{-1}.

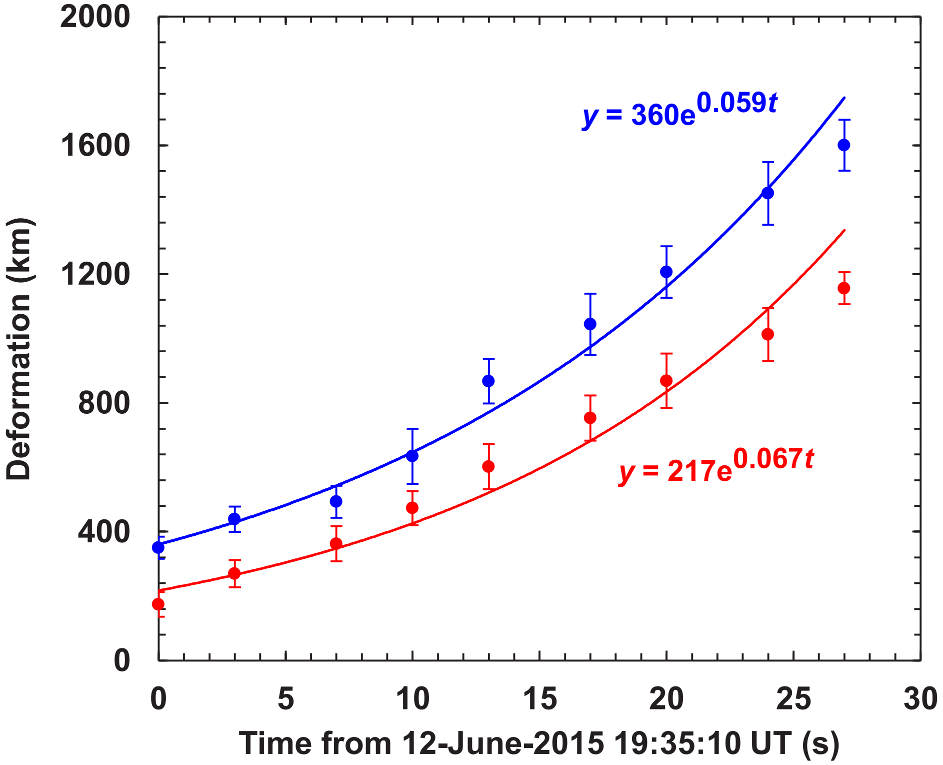

Supplementary Figure 3 | The growth rate of the KHI. We choose two positions on the jet and measure the distortions over time for ten times. The blue and red points plot the average values and the error bars indicate the standard deviations. The blue and red lines denote corresponding fitted curves and the fitted equations are displayed beside.

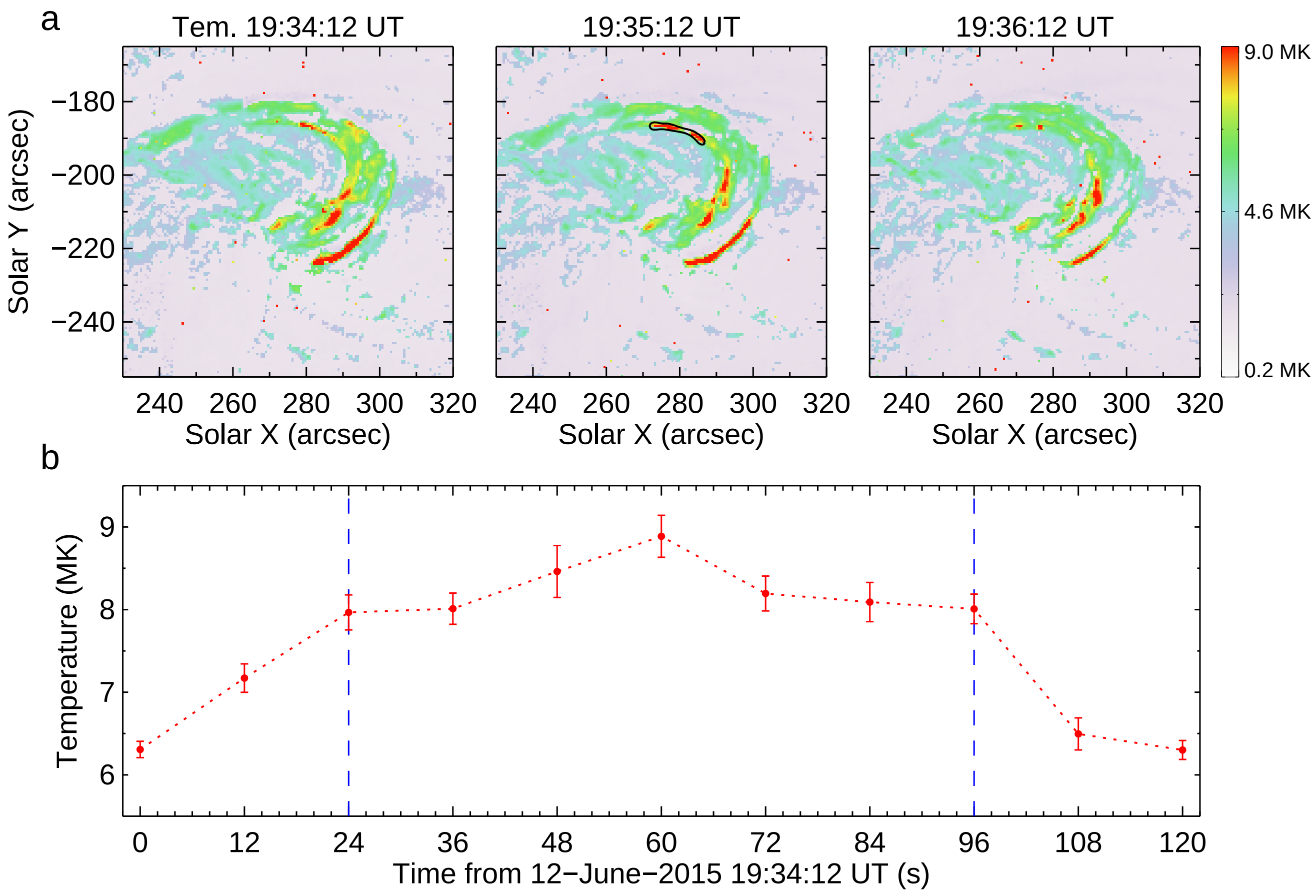

Supplementary Figure 4 | Temperature increase caused by the KHI. a plots the temperature variation derived from the wavelengths of 94 Å, 131 Å, 171 Å, 193 Å, 211 Å and 335 Å. We choose an area (outlined by the black contour) where the KHI occurs as shown in the middle panel and calculate the average temperature over time at five instances. The average values are given in **b** (red circles) and the error bars indicate the standard deviations. The blue dashed lines denote the start and end times of the temperature increase, i.e. marking, basically the same start and end times of the KHI.

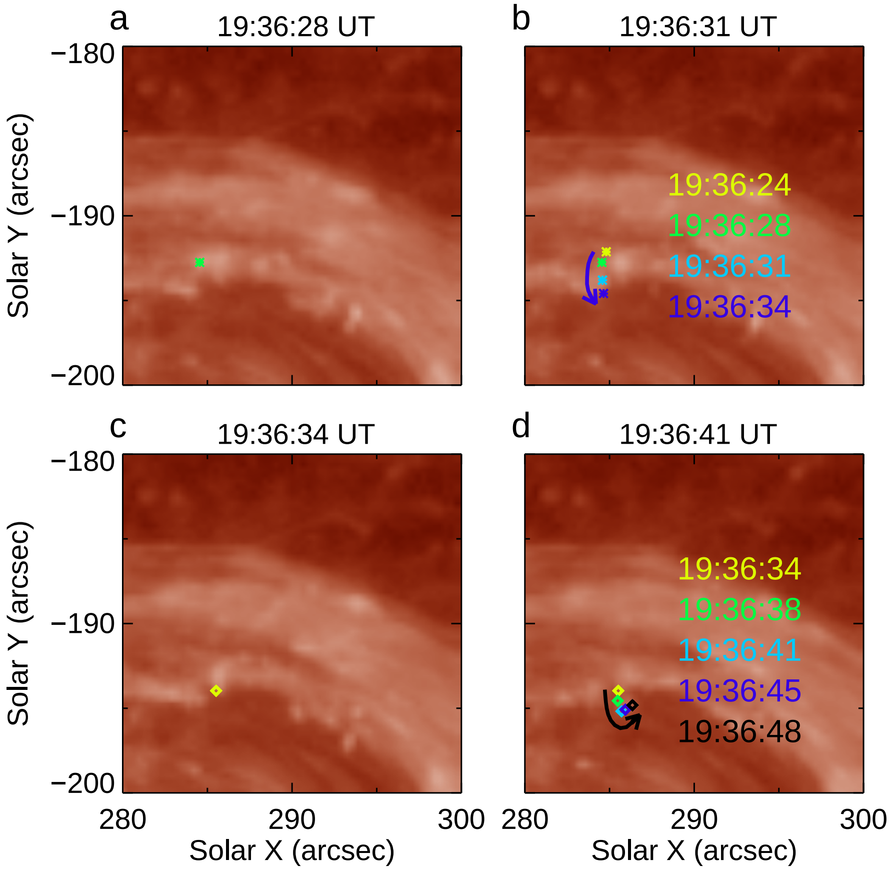

Supplementary Figure 5 | IRIS 1400 Å images displaying the rotation triggered by the KHI. The positions of the tracking points are indicated by the same symbols. The time of each position is denoted by the same symbol color and the arrows display the directions of motion.

a

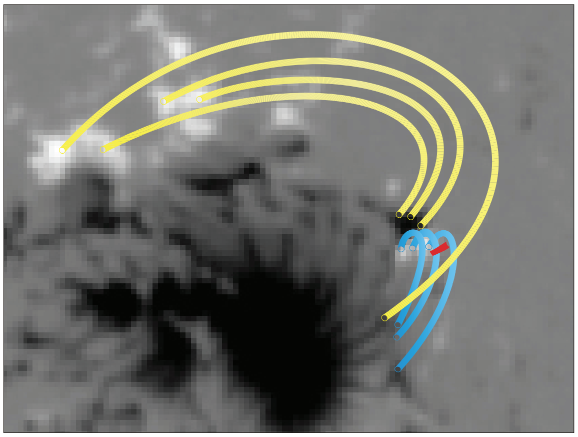

b

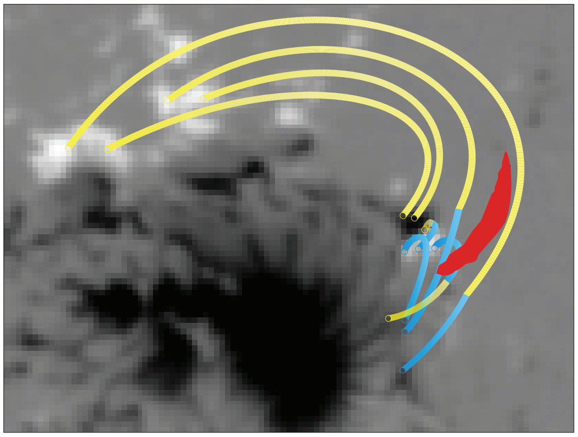

Supplementary Figure 6 | The reconnection scenario of the jet. The backgrounds of **a** and **b** are HMI LOS magnetograms at 19:25:25 UT and 19:30:40 UT, respectively. The yellow lines represent the ambient field lines. The cyan lines indicate the new emerging magnetic flux. The red patterns represent the cold filament material and the filament in **b** is contoured from the AIA 304 Å image sampled at 19:30:33 UT.

Supplementary Video 1 | Line-of-sight (LOS) magnetic field evolution. The LOS magnetic field evolution of AR 12365 in the photosphere of the Sun was obtained by SDO/HMI from 00:30 UT to 24:00 UT on June 12, 2015. The field-of-view (FOV) is $100''$ $\times$ $100''$ and the cadence of the magnetograms is 30 min. The green box at each frame indicates the approximate area where the positive emerging flux cancels with the pre-existing negative fields and the jet develops.

Supplementary Video 2 | SDO/AIA 304 Å observations displaying the evolution of the jet. The evolution of the jet was observed by the SDO/AIA from 19:24 UT to 19:50 UT on June 12, 2015. The jet was accompanied by an active penumbral filament. The FOV is $100'' \times 100''$ and the cadence of the images is 12 s. The jet and the filament are pointed out by white arrows in the images.

Supplementary Video 3 | The evolution of the jet observed by IRIS. The evolution of the jet was detected by IRIS in 1400 Å from 19:24 UT to 19:50 UT on June 12, 2015. The FOV is $100'' \times 100''$ and the cadence of the images is 17 s. The green boxes outline the brightnings at the tail of the jet and the blue dotted curves denote a "cavity" within the jet.

Supplementary Video 4 | IRIS 1400 Å observation of the KHI in the jet. The development of the KHI was detected by IRIS in 1400 Å in the jet. Two upward flows crossed through the left boundary of the jet successively. Blue arrows in the frames point out the first flow "F1" from 19:33:15 UT to 19:33:45 UT and the second flow "F2" from 19:34:33 UT to 19:35:10 UT. The left boundary of the jet was smooth at first and developed into sawtooth pattern, after the second flow passed by. The black curves show the change of the boundary. The FOV is $30'' \times 30''$ and the cadence of the images is 3 s.

Supplementary Video 5 | AIA 304 Å observation of the KHI in the jet. The left boundary of the jet was smooth at first and developed into sawtooth pattern, after the

second flow passed by. The black curves show the change of the boundary. The FOV is $40''$ $\times$ $40''$ and the cadence of the images is 12 s.
